# Supplementary material for: Evaluating the psychosocial benefits of mindfulness-based art therapy for breast cancer patients: a preliminary study
Source: BMC Psychol. 2026 Aug 4;14:1568. doi: 10.1186/s40359-026-05154-5 (PMC13721674; doi:10.1186/s40359-026-05154-5)
Supplement: Supplementary file 1 [file 40359_2026_5154_MOESM3_ESM.pdf]

# Similarity Report

*Yazar* Volkan DEMİR

---

**Gönderim Tarihi:** 29-Ağu-2025 07:20ÖS (UTC+0300)
**Gönderim Numarası:** 2571149788
**Dosya adı:** FULL_TEXT.docx (75.81K)
**Kelime sayısı:** 7549
**Karakter sayısı:** 45549

**Evaluating the Psychosocial Benefits of Mindfulness-Based Art Therapy for Breast Cancer Patients: A Preliminary Study**

Volkan Demir[1], Gelisim University, Turkey Istanbul; demir.volkan@outlook.com/ ORCID: 0000-0002-8148-8647

Esra Savaş[2], Yeditepe University, Turkey Istanbul; dresrasavas@gmail.com; ORCID: 0000-0002-8178-6804

Corresponding author: Volkan Demir[1], Gelisim University, Turkey Istanbul; demir.volkan@outlook.com/ ORCID: 0000-0002-8148-8647

**Evaluating the Psychosocial Benefits of Mindfulness-Based Art Therapy for Breast Cancer Patients: A Preliminary Study**

**Abstract**

This preliminary study evaluates the psychosocial benefits of mindfulness-based art therapy (MAT) for breast cancer patients. A quasi-experimental design was used with 14 participants in the experimental group and 15 in the control group. The experimental group attended eight weekly 3-hour MAT sessions, while the control group attended a single 3-hour seminar on coping strategies during cancer. Pre- and post-intervention measures assessed coping attitudes, cancer empowerment, grief symptoms, and general health. Data analysis involved the Mann-Whitney U test and the Wilcoxon signed rank test. The results indicated that MAT significantly improved coping attitudes, cancer empowerment, and grief symptoms but did not impact general health. These findings suggest that MAT holds potential psychosocial benefits for breast cancer patients, particularly in enhancing cancer empowerment, coping strategies, and reducing grief symptoms. Future research should focus on more comprehensive trials to systematically validate the efficacy of MAT in oncology settings.

**Keywords:** Breast Cancer, Cancer Empowerment, Grief, Group Therapy, Mindfulness-Based Art Therapy, Psycho-Oncology.

## Introduction

Art therapy is a psychological support method that employs artistic materials under the guidance of a trained art therapist to alleviate individuals' physical and psychological difficulties, enhance their motivation for life and personal goals, and help them cope with daily stress [1]. In the literature, it has been noted that traumatized individuals often struggle to verbally express their intense emotions, and art proves especially valuable for those

diagnosed with serious illnesses [2]. Art therapy can be applied as an independent treatment or integrated into various psychotherapeutic approaches, such as cognitive-behavioral, humanistic, or psychoanalytic therapy. By incorporating art therapy into their therapeutic frameworks, mental health professionals enable patients to express intense thoughts and feelings through creative techniques [3].

Mindfulness-based art therapy (MAT) is theoretically grounded in self-regulation theory. This theory suggests that an individual's development depends on the extent to which they are aware of their own abilities and motivated to improve them. The primary goal of MAT is to cultivate awareness and acceptance of life challenges by enhancing an individual's capacity to acknowledge and integrate both internal and external experiences [4]. Programs designed for this purpose typically combine three core components: standard mindfulness-based stress reduction techniques, art therapy practices, and supportive group therapy [5].

The literature indicates that art and meditation practices can reduce psychological symptoms [6–8], support psychosocial adjustment [9], and enhance overall well-being [10–11]. Research further suggests that mindfulness-based art therapy improves both physical and psychological well-being [12]. Cancer, as a unique physical illness, presents significant psychosocial challenges [13]. In this context, Monti et al. (2004) demonstrated that mindfulness-based art therapy is an effective method for improving the mental and physical health of breast cancer patients [14].

Breast cancer is one of the most common cancers among women worldwide (WHO, 2020). Statistics indicate that it accounts for approximately 15% of all cancer-related deaths in women [15]. Its fatal nature, combined with the risk of recurrence despite treatment, often generates high levels of anxiety and fear. These psychological responses may be accompanied by psychiatric disorders, further complicating the illness experience. The

coexistence of medical and psychiatric conditions makes it more difficult for patients to adapt to treatment, and adversely affects both the treatment process and the course of the disease. The most common psychiatric disorders observed in cancer patients are adjustment disorder, major depression, and anxiety disorders [16–17]. It is also important, however, to distinguish grief disorder, which presents with symptoms similar to those of adjustment disorder and major depressive disorder [18–19]. Individuals with grief disorder face elevated risks of impaired mental and physical functioning, as well as increased rates of suicide and self-harm. They are also more likely to experience difficulties in psychosocial adjustment [20]. For this reason, it is essential to differentiate grief disorder from major depression and adjustment disorder in cancer patients, and to identify it accurately through diagnostic evaluation [21].

Empowerment during the cancer process is closely linked to the level of control patients have over their illness, treatment, and care [22–23]. Strategies to promote empowerment may include enhancing self-confidence, encouraging a more active role in self-care, providing information about the disease and its course, and fostering acceptance and social support. The overarching aim is to increase patients' functionality, enable them to engage in daily life, meet their basic needs independently, and strengthen their social networks [22–24]. Achieving these goals requires that patients' coping strategies be both functional and solution-oriented [25].

Coping strategies encompass a wide range of techniques that individuals begin to develop from birth, selecting those that prove effective in certain situations and modifying or diversifying them when they no longer suffice. Under heightened stress, however, previously relied-upon strategies may become dysfunctional [26]. For individuals facing cancer for the first time, coping resources may be particularly limited. Mindfulness-based art therapy techniques represent one approach that can support cancer patients in managing stressful experiences. Monti (2004) found that the use of mindfulness-based art therapy improved both the

psychological and social well-being of breast cancer patients [14]. Nevertheless, no prior study has examined the effectiveness of mindfulness-based art therapy in enhancing cancer empowerment, improving coping attitudes, reducing grief symptoms, or influencing general health among breast cancer patients. The purpose of the present study was to evaluate the effects of a mindfulness-based art therapy program on cancer empowerment, coping attitudes, grief symptoms, and general health in individuals diagnosed with breast cancer. In line with this aim, the following hypotheses were tested:

1. There was a significant difference between the pretest and posttest scores on the CopingAttitudes Assessment Scale, Cancer Empowerment Scale, Prolonged Grief Disorder Scale, and General Health Questionnaire of individuals diagnosed with breast cancer who participated in the Mindfulness-Based Art Therapy Program in favor of the posttest.

2. There was no significant difference in the pretest and posttest scores on the Coping Attitudes Assessment Scale, Cancer Empowerment Scale, Prolonged Grief Disorder Scale, or General Health Questionnaire among the individuals diagnosed with breast cancerin the control group.

3. There was a significant difference in the posttest scores on the Coping Attitudes Assessment Scale, Cancer Empowerment Scale, Prolonged Grief Disorder Scale, and General Health Questionnaire between the experimental and control groups.

## Methods

### Participants

This was a quasi-experimental study designed to examine the effectiveness of a mindfulness-based art therapy program on cancer empowerment, coping attitudes, general health, and grief symptom levels in breast cancer patients. An experimental design with a pretest-posttest

application sequence was used in this study. Two groups were formed: the experimental group and the control group. A mindfulness-based art therapy program was applied to the experimental group. The control group attended a single 3-hour seminar on coping strategies during cancer, which was conducted after the completion of the post-test scales.

To reach participants, the program was first announced through networking and social media. Of the 73 people who applied through these announcements, those who met the criteria were randomly assigned to the program. Fifteen people were assigned to the experimental group, and fifteen people were assigned to the control group. Participants were given verbal and written information about the purpose of the program and signed an informed consent form. Signed consent forms were collected from participants who chose to participate in the study. The study was conducted at the Institute for Psychosocial Wellbeing. The minimum age of the participants was 37 years, and the maximum age was 56 years. The exclusion criteria were psychotic patients, alcohol dependence, and physical, hearing, and visual disabilities that severely limited their physical activity.

**Sociodemographic**

Sociodemographic information was collected by the researchers including questions such as name, surname, telephone number, age, gender, education, employment and economic status, marital status, number of children, cohabitants, health insurance status, type, and phase of cancer [Table 1].

**Table 1** Sociodemographic characteristics of the groups

| | | Experimental Group n | Control Group n |
|---|---|---|---|
| **Age** | 35-45 | 6 | 5 |
| | 46–55 | 7 | 8 |
| | 56-65 | 1 | 2 |

| | | | |
|---|---|---|---|
| **Education** | High School | 3 | 4 |
| | Undergraduate | 10 | 9 |
| | Graduate | 1 | 1 |
| **Marital Status** | Single | 1 | 3 |
| | Married | 11 | 9 |
| | Divorced | 2 | 3 |
| **Employment** | Current employed | 10 | 9 |
| | Unemployed | 4 | 6 |
| **Cancer Stage** | Phase I | 4 | 5 |
| | Phase II | 6 | 5 |
| | Phase III | 3 | 3 |
| | Phase IV | 1 | 2 |

**Data collection tools**

**Coping Attitudes**

Coping attitudes were assessed using the Coping Attitudes Assessment Scale (COPE-R), developed by Carver, Scheier and Weintraub (1989) and its Turkish validity and reliability were assessed by Dicle et al. (2015) [27-28]. This scale, which consists of 40 items answered on a 4-point Likert scale, is scored with 5 subscales. The subscales are self-help, approach, adaptation, avoidance-avoidance, and self-punishment. The overall Cronbach's alpha coefficient of internal consistency was 0.97, and the Cronbach's alpha coefficients of the subscales were 0.96 for "self-help", 0.98 for "approach", 0.98 for "adaptation", 0.98 for "avoidance-avoidance", and 0.98 for "self-punishment". A high score is interpreted as a high level of coping and a low score is interpreted as a low level of coping.

**Cancer Empowerment**

Cancer empowerment was assessed using the Cancer Empowerment Scale (CES), developed by Van den Berg et al. (2013) and a Turkish validity and reliability study was conducted by

Yılmaz-Karabulutlu et al. (2021) [22-29]. This 5-point Likert-type scale, in which 40 items are answered with 4 subscales, aims to determine the areas where patients in the cancer process are empowered. The 4 subscales are personal power, social support, health services, and community support. The total Cronbach's alpha internal consistency coefficient was .93 for the personal power subscale, .87 for the personal power subscale, 0.95 for the social support subscale, .87 for the health subscale, and .94 for the social support subscale. The higher the total score obtained from the scale is, the more empowered the patient is. The determination of which subscale is empowered is based on the scores obtained from the problems in that subscale. Personal power (19 items; 3,4,10,12,13,15,18,20,21,26,30,31,32,33,34,35,37,38,40), social support (9 items; 5,7,8,9,16,24,25, 27,39), health services (6 items; 1,6,14,17,22,29) and community support (6 items; 2,11,19,23,28,36).

**Grief symptoms**

Grief symptoms were assessed using the The Prolonged Grief Disorder Scale (PG-12) developed by Prigerson et al. (2008), which was translated into Turkish by Gökler et al (2017) [30-31]. The Cronbach's alpha internal consistency coefficient was found to be 0.88. This 4-item, 12-item Likert-type scale helps to assess the emotional experiences and grief reactions of cancer patients regarding the losses caused by the disease. An increase in the total score obtained from the scale is interpreted as an increase in grief symptoms.

**General Health**

The General Health Survey-28 (GHS-28) developed by Goldberg (1972) was translated into Turkish by Kılıç (1996) [32-33]. The Cronbach's alpha reliability coefficient was found to be 0.94. A 4-point Likert-type scale consisting of 28 items was used in this study. The 28 items have 4 subscales, and each of them has 7 items. These subscales are somatic symptoms, anxiety

and insomnia, impairment in social functions, and major depression. The lowest possible score is 0, and the highest is 28. Scores of 0- 4 are interpreted as 'normal' mental health, and scores of 5 and above are interpreted as 'at risk'

**Intervention**

Based on a review of the literature describing established mindfulness-based art therapy programs, researchers developed the Mindfulness-based Art Therapy program (MAT) [3-35-36]. The main goal is to increase participants' awareness of their feelings and thoughts, enable them to express them verbally and through drawing, and help them cope by facilitating perceptual acceptance of the resulting expressions. The recommended ingredients are white or colored paper, writing utensils, crayons, markers, clay, glue, and, depending on personal preference, all kinds of art materials such as feathers, glitter, sequins, beads, ribbons, string, buttons, cotton, and pieces of fabric.

The MAT program for breast cancer patients included eight 180 min. (3-hour) sessions held weekly. The sessions were completed in a private room of the psychosocial wellbeing institute which has a view of the sea. Each session in the MAT consisted of an opening, middle, and closing section and was designed to encourage participants to cultivate a practice of expressing their emotions and thoughts with accepting support from other participants. The opening section included talking about the purpose of that session and general feelings on that day. The middle section included the practice of art therapy techniques. The closing section was about reflecting on their creations and analyzing each other’s creations, as well as encouraging positive and empowered words.

The mindfulness-based art therapy program was led by two professional PhD psychologists, authors VD and ES. One of the leaders was a researcher (PhD, psychologist) who specializes

in art therapy, and has **8 years** of teaching and therapist experience. The other leader was another researcher (PhD, clinical psychologist) who has an MSc in psycho-oncology, and has 15 years of teaching and therapist experience.

To increase participation and accessibility, the researchers implemented several strategies. One was to set a cutoff time of 9 p.m. in the evening, after rush hour, so that people could make it to the program. To facilitate recall, regular reminders for each session were sent the day before the program using the participant's preferred method of communication (phone, text, or email). If participants missed sessions due to emergencies or other health issues, researchers provided additional support sessions. An outline of the main themes of each session is shown in Table 2.

In addition to the session structure and materials, the MAT program was guided by a therapeutic stance characterized by empathy, non-judgmental acceptance, and supportive facilitation of group sharing. Facilitation methods included the use of open-ended questions, reflective dialogue, and opportunities for both individual and collective expression. Each session balanced mindfulness and art components by beginning with a short mindfulness exercise (e.g., breathing, body awareness, guided meditation), continuing with an art-making activity, and concluding with group reflection and sharing. Group processes were managed by establishing a safe and confidential environment, promoting mutual respect, and ensuring equal participation. Emotional safety was prioritized by validating participants' feelings, monitoring potential distress, and providing supportive redirection or additional resources when necessary. This framework ensured that participants could engage deeply with the intervention while maintaining a secure and supportive therapeutic setting.

**Table 2** Overview of the MAT Program for Breast Cancer Patients

| Session Number | Session theme | Content overview |
|---|---|---|
| **I** | Increasing awareness | *'Draw a flower'* |

| | | |
|---|---|---|
| | on personal experience and goal setting | Focus on emotions related with used colors and shapes. |
| **II** | Accepting feelings in the here and now without judgment | *Meditation<br>*'*Write a poem describing yourcurrent feelings, thoughts, and experiences.*<br>*Share your emotions throughout the poem. |
| **III** | Connecting with own bodies. | *Body awareness exercise. *'*Determine a color for your current body sensations and to paint a landscape picture using the colors you determined.*<br>*Share your emotions through the colors used and the pictures made. |
| **IV** | Consciously tracing body sensations. | Close your eyes and focus on your body.<br>*Where in your body do you feel the most challenging emotions? Now,* transform the challenging emotions you experienced in your bodies into a sculpture using clay. Finally, write a dialog in the form of "me and the sculpture" including your feelings and thoughts about the sculpture you made. |
| **V** | Disease management and problem solving. | What do illness and being sick mean to you? Now, draw a picture describing your illness.<br>Next is lets discuss '*what does your well- being mean to you?*<br>*Now,* create a picture describing this state of well-being.<br>*Lets' discuss e*motions and thoughts through your pictures. |
| **VI** | Concretizing the symptoms of illness into a form that enables participants to change the way they relate to them. | Create a sculpture representing your illness using clay.<br>To describe the physical, cognitive, and emotional reactions of the anxiety caused by the disease, *'Write a dialog in the form of "me and the sculpture" including your beliefs about the disease.* |
| **VII** | Integration and acceptation of the disease and emotions experience. | Explain the nature of "masks."”<br>*Create a mask to create a visual representation of the support and compassion you had shown yourselves before the illness.*<br>*Now, create a mask to create a visual representation of the support and compassion you had shown yourselves "here and now" after he illness.*<br>*'Create a dialog between the two masks'* to better understand and manage their emotions and thoughts, to adapt self-compassion to your daily lives, and to accept challenging experiences instead of being judgmental about them. |
| **VIII** | Helping clients to accept themselves with their positive and negative characteristics and develop feelings of self-worth and self-understanding. | *Read Mevlana Jalaluddin Rumi's poem titled 'Guest House'. What are the prominent emotions and thoughts in this poem? What does this poem make you feel?*<br>Now, write a letter to Mevlana with the theme of acceptance in response to your feelingsrevealed through the poem with reference to. |

**Statistical analysis**

To analyze the research data, the Mann- Whitney U test and Wilcoxon signed- rank test were used as nonparametric tests with the SPSS 27.0 program.

**Ethical aspects of the study**

Ethical approval for this study was obtained from the Yeditepe University Ethics Committee on 20.02.2024 with decision 48/2024.

**Results**

In this study, the effects of mindfulness-based art therapy on the empowerment, coping attitudes, general health, and grief response levels of individuals diagnosed with breast cancer who participated in the program were examined. The results of the outcomes are reported below and presented in Table 3-6.

**Table 3** Comparison of Pretest Scores for the Coping Attitudes Assessment Scale, Cancer Empowerment Scale, Prolonged Grief Disorder Scale, and General Health Questionnaire

| Outcome | Groups | n | Rank mean | Rank sum | U | p |
|---|---|---|---|---|---|---|
| **CAS** | Experiment | 14 | 15,82 | 221,50 | 93,50 | ,615 |
| | Control | 15 | 14,23 | 213,50 | | |
| **CES** | Experiment | 14 | 16,43 | 230,00 | 85,00 | ,382 |
| | Control | 15 | 13,67 | 205,00 | | |
| **PGDS** | Experiment | 14 | 14,04 | 196,50 | 91,50 | ,555 |
| | Control | 15 | 15,90 | 238,50 | | |
| **GHQ** | Experiment | 14 | 1,50 | 1,50 | ,50 | ,480 |
| | Control | 15 | 2,25 | 4,50 | | |

Abbreviations: CAS= Coping Attitudes Scale; CES= Cancer Empowerment Scale; PG= Prolonged Grief Disorder Scale; GHQ= General Health Questionnaire

Table 3 shows that the significance level of each measurement tool was greater than 0.05. All participants in the experimental and control groups received statistically equivalent scores on the scales before starting the experimental application.

**Table 4** Pretest and Posttest Wilcoxon Signed Rank Test Results of the Coping Attitudes Assessment Scale, Cancer Empowerment Scale, Prolonged Grief Disorder Scale, and General Health Questionnaire of the Experimental Group

| | Variables | Median | %25 | %75 | X±SS | Z | p |
|---|---|---|---|---|---|---|---|
| **CAS** | **Pretest** | 88,00 | 78,25 | 92,25 | 85,357±9,09 | -3,297 | ,001 |
| | **Posttest** | 119,00 | 107,00 | 122,75 | 114,928±10,16 | | |
| **CES** | **Pretest** | 155,50 | 144,00 | 168,75 | 156,500±18,16 | -2,075 | ,038 |
| | **Posttest** | 166,00 | 150,00 | 176,00 | 163,500±19,64 | | |
| **PGDS** | **Pretest** | 27,50 | 20,75 | 35,25 | 28,357±9,30 | -2,390 | ,017 |
| | **Posttest** | 24,00 | 18,75 | 31,25 | 24,428±6,62 | | |
| **GHQ** | **Pretest** | 3,00 | ,75 | 4,50 | 4,285±5,84 | -1,843 | ,065 |
| | **Posttest** | 1,00 | ,00 | 4,25 | 2,928 ±4,58 | | |

Abbreviations: CAS= Coping Attitudes Scale; CES= Cancer Empowerment Scale; PG= Prolonged Grief Disorder Scale; GHQ= General Health Questionnaire

Table 4 shows that there was a statistically significant difference between the scores of the experimental group on the Coping Attitudes Assessment Scale, Cancer Empowerment Scale and Prolonged Grief Disorder Scale before and after the intervention (empowerment z=, -3,297 p<.05; coping attitudes z=,-2,075 p<.05; grief reaction z=,-2,390 p<.05). There was no statistically significant difference between the scores of the General Health Questionnaire before and after the application (general health z=, -1,843 p>.05).

**Table 5** Pretest and Posttest Wilcoxon Signed Rank Test Results of the Coping Attitudes Assessment Scale, Cancer Empowerment Scale, Prolonged Grief Disorder Scale, and General Health Questionnaire of Individuals in the Control Group

| | Variables | Median | %25 | %75 | X±SS | Z | p |
|---|---|---|---|---|---|---|---|
| **CAS** | **Pretest** | 87,00 | 78,00 | 91,00 | 84,200±7,79 | -1,006 | ,314 |
| | **Posttest** | 76,00 | 73,00 | 89,00 | 80,533±9,12 | | |
| **CES** | **Pretest** | 151,00 | 138,00 | 168,00 | 150,600±18,24 | -1,762 | ,078 |
| | **Posttest** | 128,00 | 125,00 | 164,00 | 140,000±21,01 | | |
| **PGDS** | **Pretest** | 28,00 | 24,75 | 38,00 | 30,266±8,21 | -,222 | ,825 |
| | **Posttest** | 31,00 | 23,00 | 38,00 | 30,466±8,87 | | |
| **GHQ** | **Pretest** | 4,00 | 3,00 | 11,00 | 6,800±5,36 | - ,885 | ,376 |

| | Posttest | 6,00 | 3,00 | 10,00 | 7,133 ±5,27 |
|---|---|---|---|---|---|

Abbreviations: CAS= Coping Attitudes Scale; CES= Cancer Empowerment Scale; PG= Prolonged Grief Disorder Scale; GHQ= General Health Questionnaire

Table 5 shows that there was no statistically significant difference between the pretest and posttest scores on the Coping Attitudes Assessment Scale, Cancer Empowerment Scale, General Health Questionnaire, or Prolonged Grief Disorder Scale for the control group (empowerment z=,-1,006 p>05; coping attitudes z=,-1,762 p>.05; grief reaction z=,-,222 p>.05; general health z=,-,885 p>.05).

**Table 6** Mann- Whitney U test posttest results for the groups on Coping Attitudes Assessment Scale, Cancer Empowerment Scale, Prolonged Grief Disorder Scale, and General Health Questionnaire

| | Groups | n | Rank mean | Rank sum | U | p |
|---|---|---|---|---|---|---|
| **CAS** | Experiment | 14 | 22,39 | 313,50 | 1,500 | ,000 |
| | Control | 15 | 8,10 | 121,50 | | |
| **CES** | Experiment | 14 | 19,32 | 270,50 | 44,50 | ,008 |
| | Control | 15 | 10,97 | 164,50 | | |
| **PGDS** | Experiment | 14 | 11,71 | 164,00 | 59,00 | ,045 |
| | Control | 15 | 18,07 | 271,00 | | |
| **GHQ** | Experiment | 14 | 4,07 | 28,50 | ,50 | ,083 |
| | Control | 15 | 7,50 | 7,50 | | |

Abbreviations: CAS= Coping Attitudes Scale; CES= Cancer Empowerment Scale; PG= Prolonged Grief Disorder Scale; GHQ= General Health Questionnaire

Table 6 shows that there was a statistically significant difference between the experimental and control groups posttest scores on the Coping Attitudes Assessment Scale, Cancer Empowerment Scale, and Prolonged Grief Disorder Scale (empowerment U=,1,500 p<.05, coping attitudes U=,44,50 p<.05, grief reaction U=,59,00 p<.05). Participants in the experimental group (attending the MAT) had better scores than did those in the control group. There was no statistically significant difference between the experimental group and control group participants' scores on the General Health Questionnaire (general health U=,50 p>.05).

## Discussion

The MAT program was designed by researchers for 8 sessions, specifically for breast cancer patients. In this study, researchers aimed to evaluate the effectiveness of the program on cancer empowerment, coping attitudes, general health, and grief reactions of breast cancer patients. The results showed that eight sessions of the MAT program for breast cancer patients significantly enhanced cancer empowerment, coping attitudes, and grief reactions, but had no effect on the general health status. This finding may be expected, since mindfulness-based art therapy interventions are not primarily designed to directly improve general health status. Instead, their favorable outcomes lie in reducing existential distress, supporting meaning-making, and facilitating acceptance of the illness experience, as well as managing the side effects of treatment. Such outcomes are highly individualized and may vary depending on the phase of the disease, personality structure, and the level of social support available. Despite the small sample size, the findings highlight the importance of implementing awareness-based interventions as early as possible in the illness trajectory. Such interventions may foster self-esteem, empowerment, and adaptation throughout the cancer experience. While these findings are encouraging, they should be understood within the context of an already growing body of research showing psychosocial benefits of both art therapy and mindfulness interventions with oncology patients. The present study contributes by specifically integrating these two approaches and focusing on empowerment and grief, outcomes that remain less frequently examined in the literature.

One of the key findings of this study is that the MAT program was effective in improving coping attitudes among breast cancer patients. Art therapy enables patients to integrate their physical, emotional, and spiritual experiences related to the illness into creative expression, helping them discover new solutions and develop coping strategies [37]. During the sessions, activities based on mindfulness-based art therapy techniques were observed to support patients

in becoming more aware of their thoughts, relaxing through emotional expression with artistic materials, and engaging willingly with the themes addressed. From this perspective, the present study offers an alternative approach for programs aimed at strengthening coping attitudes. The results are consistent with those of Öster et al. (2006), who reported that art therapy programs for breast cancer patients were effective in enhancing coping resources, a core component of psychological well-being during the cancer process [38]. Overall, the MAT program for breast cancer patients appears to be a feasible, safe, cost-effective, and innovative intervention for enhancing psychosocial well-being, particularly with regard to empowerment, coping attitudes, and grief reactions in oncology settings.

Another finding of our research is that attending the MAT program empowers participants in terms of intrapersonal and interpersonal strengths. One of the goals of the MAT program was to provide cancer patients with the opportunity to discover and empower themselves through the creative process. The results of the study showed that integrating creative art therapy into traditional therapeutic approaches empowers patients to freely express their feelings and thoughts without feeling restricted or pressured. Patient empowerment increases coping with the disease and self-efficacy [39]. Patient empowerment is both a process and a product, with the outcome of increasing the self-efficacy of cancer patients [40]. During the preparation of the therapy program applied in the study, group sharing, and group discussions were included by applying art therapy practices that enable individuals to establish a more flexible relationship with the symptoms of the disease rather than the difficulties caused by the disease to help individuals accept their illness, cope effectively, and improve their cognitive, social, and physical health. After these practices, it was expected that participants' levels of empowerment with cancer would increase. The analysis confirmed this expectation. One of the reasons that makes this study noteworthy is that when the literature was reviewed as part of this study, there were no studies that examined the effect of the MAT program on the

experience of cancer empowerment in individuals diagnosed with breast cancer. Therefore, these findings could not be compared to those in the literature.

One of the other findings of the study showed that attending the MAT program helps to reduce grief symptoms in breast cancer patients. The grieving process is a normal process that does not require intervention, but this finding cannot be generalized to everyone or every situation. Social norms or other obstacles might lead grieving people to feel stuck and have difficulty completing this normal process. This constriction might turn the normal grief process into a complicated grief. During these complicated grief processes, it is better to obtain support from others [41]. Getting support could be received from both professional and nonprofessional sources. Nonprofessional support sources include family members, friends, neighbors, and religious communities. Professional support sources include mental health professionals, social workers, and art therapists [42].

Art therapy is one of the approaches for healing grief symptoms [43-46]. The reason for its effectiveness is that art therapy is a channel for individuals to express their feelings about their losses and to nonverbally express their loss experiences [47-48]. In this study, group sharing was practiced using art therapy techniques such as painting, poetry, and music. During sessions, participants shared their experiences based on the here and now principle according to the current needs of the group members. It was expected that the participants' awareness of the similarity of their illness experiences to the feelings, thoughts, and experiences of other individuals would prevent their judgmental attitudes toward themselves and negative self-focus. On the other hand, it was expected that the sense of "universality," which is the most fundamental advantage of group interventions, would have a positive effect on grief symptom levels. The pretest and posttest scores on the Prolonged Grief Disorder Scale supported this expectation. This result is one of the significant contributions to the field

because no studies have examined the effects of attending a mindfulness-based art therapy program on the grief symptoms.

The other finding takes attention of this study is its lack of effect on the general health status of breast cancer patients attending to the MAT program. The literature has shown that, attending art therapy programs during a serious illness is effective in enhancing the adjustment to illness and treatment, finding meaning in life, increasing awareness of cancer and life, improving skills to express emotions and thoughts, and improving psychological well-being [38-49-50]. Additionally, attending art therapy programs helps patients reduce pain and psychological symptoms and to improve quality of life [51-54]. For example, Monti et al. (2006) conducted an eight-week mindfulness-based art therapy program for women who had cancer [5]. The results showed that attending his program decreased in the overall health level of the patients. Jalambadani et al. (2019) reported similar results with their mindfulness-based art therapy program for women with breast cancer [51]. In our study, even though there was a significant increase in the overall health level of the participants, it was not as effective as in the other studies in the literature. The reason might be that our participants were from all stages of cancer; some of them were diagnosed only, some were receiving active treatment, and some were completing their treatment processes.

The analyses of the pretest and posttest scores of the experimental and control groups revealed significant differences in terms of the scores on the Coping Attitudes Assessment Scale, Cancer Empowerment Scale, and Prolonged Grief Disorder Scale. The content of the sessions was designed to enable participants to express their cancer experiences through nonverbal communication; to increase patients' coping resources by combining their physical, emotional, and spiritual experiences related to the illness through the creative process; to accept patients' challenging experiences in the present moment through mindfulness practices; to establish a more flexible relationship with the symptoms of the

illness rather than the difficulties caused by the illness; and to establish a more flexible relationship with the symptoms of the illness. It was expected that individuals would become stronger against cancer, their coping attitudes would increase, and their grief reactions would decrease. The results of the study support these expectations.

The General Health Questionnaire scores of the experimental and control groups did not significantly differ. Although it was expected that integrating mindfulness practices with art therapy practices during these sessions would help cancer patients connect their physical, emotional, and spiritual experiences related to the cancer with art, find new ways to express their cancer experiences; adjust to life changes, illness and treatment processes; become aware of the deep emotions; and have an effect on their general health level; the results did not meet these expectations. The reason might be the illness- related differences of the participants and the structure of the program, which was once a week for a total of eight weeks. In this context, a structured short psychological intervention program based on mindfulness and art therapy approaches may not have been effective enough to show a significant difference in general health.

To summarize, this study contributes to the literature in many ways. Although psychosocial interventions are widely used, few studies have tested the effect of mindfulness-based art therapy in patients diagnosed with breast cancer. There are almost no studies reporting the effectiveness of mindfulness-based art therapy, particularly for grief symptoms, cancer empowerment, and coping skills. Therefore, the limited number of studies on this topic limits our ability to compare the results of our study. It would be useful to develop studies that test the effect of mindfulness-based art therapy based on the limitations of this study and the suggestions we have presented. Similar studies can be conducted in individuals with different cancer diagnoses and at different stages of treatment. Follow-up studies can be conducted at specified intervals to determine whether the effects of mindfulness-based art therapy are

sustained. The MAT can be enriched as a psychosocial approach by making it a routine treatment model in psycho-oncology services and with the participation of mental health professionals other than psychologists.

**Study Limitations**

The study had several limitations. Retention measures were not conducted to determine the permanence of the positive outcomes found in the research. Some of the findings may be related to the structure of the program, which was completed in 8 sessions and 180 minutes, which is not sufficient for the psychosocial needs of the patients. In this context, it would be recommended to increase the number and duration of sessions in future studies, especially for a dynamic that can change and develop within the psychosocial intervention process, such as mental health problems. Another limitation of the study is that it was not compared with an alternative psychosocial treatment program that does not include mindfulness-based art therapy practices, such as cognitive-behavioral, Gestalt, or existentialist practices. The other limitation is that our research did not use DSM diagnoses. Instead, we relied on symptom severity ratings derived from validated self-report questionnaires rather than V diagnoses. It is plausible that some of the participants in the study may have met the diagnostic criteria for the disorders listed, but they were not seeing a psychiatrist and had no knowledge or awareness of them. This may be considered a limitation. A major limitation of this study is the absence of follow-up assessments at regular intervals, such as 3 or 6 months. Although short-term effectiveness of the MAT program was observed, it remains unclear whether these improvements in empowerment, coping, and grief are sustained over time. This significantly limits the ability to evaluate the long-term stability of the intervention's outcomes. Future research should incorporate longitudinal follow-up sessions to determine whether the benefits of MAT persist and to better understand its potential as an ongoing psychosocial support model for breast cancer patients. Another important limitation is the very small sample size, which represents a

major restriction on the generalizability and statistical power of the findings. The results should therefore be interpreted with caution. Nevertheless, even with this small sample, the study provides preliminary evidence that underscores the importance of early implementation of awareness-based interventions in the cancer trajectory, as such interventions may foster self-esteem, empowerment, and adaptation. In addition, the control condition, which consisted of a single coping seminar, was not equivalent in length or intensity to the eight-session MAT program. This discrepancy makes it difficult to attribute group differences solely to the specific effects of MAT. The exclusive reliance on self-report instruments, without diagnostic interviews, clinician ratings, or objective behavioral assessments, further limits the depth of the findings. Moreover, the absence of effect size reporting constrains the ability to interpret the clinical significance of the results. In addition, specific aspects of the intervention design and dosage, as well as the non-equivalent control condition, may have influenced the results. These methodological issues should be taken into account when interpreting the findings. A larger and more diverse sample size with a randomized design and follow-up measurement after 6 months of intervention would contribute to the robustness and applicability of the study's conclusions. These limitations should be addressed in future studies.

**Clinical implications**

The MAT appears to be a feasible and flexible intervention for breast cancer patients to enhance coping, cancer empowerment, and reduce grief symptoms.

**Conclusions**

The MAT showed positive effects on improving coping, cancer empowerment, and reducing grief symptoms after treatment. These results provide compelling evidence for the positive

impact of the MAT on the psychological well-being of breast cancer patients at different stages of the disease. The findings underscore the potential of MAT as a valuable therapeutic approach for alleviating psychological distress in women with breast cancer.

**Abbreviations**

**MAT**: Mindfulness-Based Art Therapy

**CAS**: Coping Attitudes Scale

**CES**: Cancer Empowerment Scale

**PGDS**: Prolonged Grief Disorder Scale

**GHQ**: General Health Questionnaire

**SPSS**: Statistical Package for the Social Sciences

**DSM-V**: Diagnostic and Statistical Manual of Mental Disorders, Fifth Edition

**Declarations**

**Funding:** The study has no funding.

**Conflicts of interest/Competing interests:** The authors declare no competing interests.

**Ethics approval:** The study was approved by the Yeditepe University Research Ethic Committee (decision number 20.02.2024-48/2024). Informed consent was obtained from all individual participants included in the study. The research was conducted in accordance with the Declaration of Helsinki.

**Consent to participate:** Written informed consent for publication of their details was obtained from the study participants.

**Consent for publication** Not applicable.

**Availability of data and materials**: Data available on request due to privacy/ethical restrictions. The data that support the findings of this study are available on request from the corresponding author. The data are not publicly available due to privacy or ethical restrictions.

**Authors' contributions:** VD: investigation, conceptualization, supervisor, participation follow up, writing—review and editing.

ES: investigation, conceptualization, supervisor, participation follow up, data duration, draft preparation, formal analysis, writing,—review and editing

All authors read and approved the final manuscript.

## REFERENCES


1. Potash, J. S. (2019). Art Therapy Gold: American Art Therapy Association at 50. *Art Therapy*, *36*(3), 113-114. https://doi.org/10.1080/07421656.2019.1660134
2. Aydın, B. (2012). Medical art therapy. *Psikiyatride Güncel Yaklaşımlar*, *4*(1), 69-83. https://doi:10.5455/cap.20120405
3. Rappaport, L. (2009). *Focusing-oriented art therapy: Accessing the body's wisdom and creative intelligence.* Jessica Kingsley Publishers.
4. Monti, D. A., & Peterson, C. (2003). Mindfulness-based art therapy (MAT). *Integrative Cancer Therapies*, *2*(1), 81-83.
5. Monti, D. A., Peterson, C., Kunkel, E. J. S., Hauck, W. W., Pequignot, E., Rhodes, L., & Brainard, G. C. (2006). A randomized, controlled trial of mindfulness- based art therapy (MAT) for women with cancer. *Psycho-Oncology: Journal of the Psychological, Social and Behavioral Dimensions of Cancer, 15*(5), 363-373. https://doi.org/10.1002/pon.988
6. Bamber, M. D., & Schneider, J. K. (2016). Mindfulness-based meditation to decrease stress and anxiety in college students: A narrative synthesis of the research. *Educational Research Review*, *18*, 1-32.
7. Eaton, J. and Tieber, C. (2017). Effects of coloring on anxiety, mood, and perseverance. *Art Therapy*, *34*(1), 42-46. https://doi.org/10.1080/07421656.2016.1277113
8. Sundquist, J., Lilja, Å., Palmer, K., Memon, A. A., Wang, X., Johansson, L. M. and Sundquist, K. (2015). Mindfulness group therapy in primary care patients with depression, anxiety, stress, and adjustment disorders: a randomized controlled trial. *The British Journal of Psychiatry*, *206*(2), 128-135. https://doi.org/10.1192/bjp.bp.114.150243
9. Henderson, V. P., Massion, A. O., Clemow, L., Hurley, T. G., Druker, S., & Hébert, J. R. (2013). A randomized controlled trial of mindfulness-based stress reduction in women with early-stage breast cancer receiving radiotherapy. *Integrative Cancer Therapies*, *12*(5), 404-413. https://doi: 10.1177/1534735412473640.
10. Prazak, M., Critelli, J., Martin, L., Miranda, V., Purdum, M., Powers, C. (2012). Mindfulness and its role in physical and psychological health. *Applied Psychology: Health and Well-Being*, *4*(1), 91-105. https://doi: 10.1111/j.1758-0854.2011.01063.x

11. Slayton, S. C., D'Archer, J., & Kaplan, F. (2010). Outcome studies on the efficacy of art therapy: A review of findings. *Art therapy*, *27*(3), 108-118. https://doi.org/10.1080/07421656.2010.10129660
12. Beerse, M. E., Van Lith, T., & Stanwood, G. (2020). Therapeutic psychological and biological responses to mindfulness-based art therapy. *Stress and Health*, *36*(4), 419-432. https://doi.org/10.1002/smi.2937
13. Elbi, H. (2001). Cancer and depression. *Psychiatry World*, *5*(1), 5-10.
14. Monti, D. A. (2004). Mindfulness-based art therapy: results from a two-year study. *Psychiatric Times*, *21*(8), 63–63.
15. Siegel, R. L., Miller, K. D., & Jemal, A. (2020). Cancer Statistics, 2020. *CA*: *A Cancer Journal for Clinicians*, *70*(1), 7-30. https://doi.org/10.3322/caac.21590
16. Philip, E. J., Merluzzi, T. V., Zhang, Z., & Heitzmann, C. A. (2013). Depression and cancer survivorship: importance of coping self-efficacy in post-treatment survivors. *Psycho-Oncology*, *22*(5), 987-994. https://doi.org/10.1002/pon.3088
17. Walker, J., Hansen, C. H., Martin, P., Symeonides, S., Ramessur, R., Murray, G., & Sharpe, M. (2014). Prevalence, associations, and adequacy of treatment of major depression in patients with cancer: a cross-sectional analysis of routinely collected clinical data. *The Lancet Psychiatry*, *1*(5), 343-350. https://doi.org/10.1016/S2215-0366(14)70313-X
18. Jacobsen, J. C., Zhang, B., Block, S. D., Maciejewski, P. K., & Prigerson, H. G. (2010). Distinguishing symptoms of grief and depression in a cohort of patients with advanced cancer *Death Studies*, *34*(3), 257–273. https://doi.org/10.1080/07481180903559303
19. Lacey, J. (2011). Recognizing grief in oncology patients and their caregivers. *Journal of Supportive Oncology*, *5*(9), 183.
20. Mystakidou, K., Tsilika, E., Parpa, E., Athanasouli, P., Galanos, A., Anna, P., & Vlahos, L. (2009). Illness-related hopelessness in advanced cancer: influence of anxiety, depression, and preparatory grief. *Archives of Psychiatric Nursing*, *23*(2), 138-147. https://doi.org/10.1016/j.apnu.2008.04.008
21. Gökler-Danışman, I., Yalçınay-İnan, M., & Yiğit, İ. (2017). Experience of grief by patients with cancer in relation to perceptions of illness: The mediating roles of identity centrality, stigma-induced discrimination, and hopefulness. *Journal of psychosocial oncology*, *35*(6), 776-796.

22. Van den Berg, S. W., Van Amstel, F. K. P., Ottevanger, P. B., Gielissen, M. F., & Prins, J. B. (2013). Cancer empowerment questionnaire: Psychological empowerment of breast cancer survivors. *Journal of Psychosocial Oncology*, *31*(5), 565–583. https://doi.org/10.1080/07347332.2013.825361
23. Marzorati C, Bailo L, Mazzocco K, Pravettoni G (2018). Empowerment from patient and caregiver perspectives in cancer care. *Health psychology open*, *5*(2), 2055102918815318. https://doi: 10.1177/2055102918815318
24. Jorgensen, C. R., Thomsen, T. G., Ross, L., Dietz, S. M., Therkildsen, S., Groenvold, M., ... Johnsen, A. T. (2018). What facilitates "patient empowerment" in cancer patients during follow-up: A qualitative systematic literature review. *Qualitative Health Research*, *28*(2), 292-304. https://doi.org/10.1177/1049732317721477
25. Aydogan, U., Doganer, Y. C., Komurcu, S., Ozturk, B., Ozet, A., & Saglam, K. (2016). Coping attitudes of cancer patients and their caregivers and quality of life of caregivers. *Indian journal of palliative care*, *22*(2), 150. https://doi.org/10.4103/0973-1075.179598
26. Folkman, S., & Moskowitz, J. T. (2004). Coping: Pitfalls and promise. *Annual Review of Psychology*, *55*, 745–774. https://doi.org/10.1146/annurev.psych.55.090902.141456
27. Carver, C. S., Scheier, M. F., & Weintraub, J. K. (1989). Assessing coping strategies: a theoretically based approach. *Journal of personality and social psychology*, *56*(2), 267. https://doi.org/10.1037/0022-3514.56.2.267
28. Dicle, A. N. and Ersanli, K. (2015). Turkish Standardization and Validity and Reliability Studies of Cope *Asos Journal, The Journal of Academic Social Science*, *16*, 111–26.
29. Yilmaz Karabulutlu E., Bahçecioglu Turan, G. & Karaman S. (2021). Turkish validity and reliability study of the cancer empowerment scale. *Psychiatria Danubina, 33*(Suppl. 13), 314-320.
30. Prigerson, H. G., Vanderwerker, L. C., & Maciejewski, P. K. (2008). *Prolonged Grief Disorder Scale--Patient Form* [Database record]. APA PsycTests. https://doi.org/10.1037/t67680-000
31. Gökler-Danişman, I., Yalcinay, M., & Yildiz, N. (2017). Measuring Grief Symptoms in Cancer Patients: The Reliability and Validity Study of the Turkish Version of Prolonged Grief Disorder Scale. *Turk Psikiyatri Dergisi*, *28*(3).
32. Goldberg, P. (1972). Detection of psychiatric illness using questionnaire *Maudsley monograph*. 252–7.

33. Kılıç C. (1996). General Health Questionnaire: Validity and Reliability Study. *Turkish Journal of Psychiatry. 2007;7(1):3*-9.
34. Demir, V. (2022). Sanat terapisi. *Temel Psikoloji Kuramları ile Dışavurumcu Sanatın Bütünleştirilmesi. Ankara: Nobel Yayınları*.
35. Jang, S. H., Kang, S. Y., Lee, H. J., Lee, S. Y. (2016). Beneficial effect of mindfulness-based art therapy in patients with breast cancer-a randomized controlled trial. *Explore*. 2016;12(5):333-340. https://doi.org/10.1016/j.explore.2016.06.003
36. Savas, E . (2022). *Cognitive Behavioral Therapy Based Stress Management Program During Cancer Process*. Nobel Publishers.
37. Horovitz, E. G. (2017). *Spiritual art therapy: An alternate path*. Charles C Thomas Publisher
38. Öster, I., Svensk, A. C., Magnusson, E. V. A., Thyme, K. E., Sjõdin, M., Åström, S., & Lindh, J. (2006). Art therapy improves coping resources: a randomized, controlled study among women with breast cancer. *Palliative & supportive care*, *4*(1), 57-64.
39. Teymouri, F., Alhani, F., & Kazemnejad, A. (2017). The effect of family-centered empowerment model on self-efficacy and self-esteem of the children with asthma.
40. Ziegler, E., Hill, J., Lieske, B., Klein, J., dem, O. V., & Kofahl, C. (2022). Empowerment in cancer patients: does peer support make a difference? A systematic review. *Psycho-Oncology*, *31*(5), 683-704. https://doi.org/10.1002/pon.5869
41. Arslan, B. S., & Buldukoglu, K. (2019). Grief Support Programs implemented to reduce the effects of grief on family *Guncel Yaklasimlar/Current Approaches to Psychiatry*, *11*(3), 402–418. doi:10.18863/pgy.444297
42. Steiner, C. S. (2006). Grief Support Groups Used by Few: Are Bereavement Needs Being Met?. *Journal of Social Work in End-of-Life & Palliative Care*, *2*(1), 29-53. https://doi.org/10.1300/J457v02n01_04
43. Irwin, H. J. (1991). Depiction of loss: Use of clients' drawings in bereavement counseling. *Death Studies*, *15*(5), 481–497. https://doi.org/10.1080/07481189108252774
44. 44. Mango, C. (1992). Emma: Art therapy illustrating personal and universal images of loss. *OMEGA-Journalof Death and Dying*, *25*(4), 259-269. https://doi.org/10.2190/B8U4-3UQ4-XYX6-G0JC
45. Schimmel, B. F., & Kornreich, T. Z. (1993). Use of art and verbal processes with recently widowed individuals. *American Journal of Art Therapy*, *31*(3), 91-97.

46. Schut, H. A. W., De Keijser, J., Van den Bout, J., & Stroebe, M. S. (1996). Cross-Modality Grief Therapy: Description and assessment of a new program. 4.4 *Journal of Clinical Psychology*, *52*(3), 357-365. https://doi.org/10.1002/(SICI)1097-4679(199605)52:3<357::AID-JCLP14>3.0.CO;2-H

47. 47. Davis, C. B. (1989). Use of art therapy and group processes with grieving children. *Issues in the Comprehensive Pediatrics Nursing,* *12*(4), 269-280. https://doi.org/10.3109/01460868909026834

48. Bardot, H. (2008). Expressing the inexpressible: The resilient healing of client and art therapist. *Art Therapy*, *25*(4), 183-186. https://doi.org/10.1080/07421656.2008.10129547

49. Ponto, J. A. (2003). Stories of breast cancer through art. *Number 6/2003*, *30*(6), 1007-1013. doi: 10.1188/03.ONF.1007-1013

50. 50. Nainis, N. A. (2008). Approaches to art therapy for cancer inpatients: Research and practice considerations. *Art therapy*, *25*(3), 115-121. https://doi.org/10.1080/07421656.2008.10129597

51. 51. Jalambadani Z, Borji A, Bakaeian M. 2019. Examining the effect of mindfulness-based art therapy (MAT) on the stress and lifestyle of pregnant Iranian women *Journal of Obstetrics and Gynaecology,* (2), 1-5. https://doi.org/10.1080/01443615.2019.1652889

52. Joshi, A. M., Mehta, S. A., Pande, N., Mehta, A. O., and Randhe, K. S. (2021). Effect of mindfulness-based art therapy (MAT) on psychological distress and spiritual well-being in patients with breast cancer undergoing chemotherapy. *Indian Journal of Palliative Care*, *27*(4), 552. https://doi.org/10.25259/IJPC_133_21

53. Meghani, S. H., Peterson, C., Kaiser, D. H., Rhodes, J., Rao, H., Chittams, J., & Chatterjee, A. (2018). A pilot study of a mindfulness-based art therapy intervention in outpatients with cancer. *American Journal of Hospice and Palliative Medicine®*, *35*(9), 1195-1200. https://doi.org/10.1177/10499091187603

54. Prioli, K. M., Pizzi, L. T., Kash, K. M., Newberg, A. B., Morlino, A. M., Matthews, M. J., Monti, D. A. (2017). Costs and effectiveness of mindfulness-based art therapy versus standard breast cancer support group for women with cancer. *American Health & Drug Benefits*, *10*(6), 288.

*Data citation:* Demir, V & Savaş, E.; 2024; A Pilot Study of Mindfulness Based Art Therapy for Breast Cancer Patients: A Cancer Empowerment Program; PsychArchieves; Persistent identifier (e.g. DOI).

## Similarity Report

ORİJİNALLİK RAPORU

| %12 | %9 | %8 | % |
|---|---|---|---|
| BENZERLİK ENDEKSİ | İNTERNET KAYNAKLARI | YAYINLAR | ÖĞRENCİ ÖDEVLERİ |

BİRİNCİL KAYNAKLAR

| | | |
|---|---|---|
| 1 | dergipark.org.tr<br>İnternet Kaynağı | %2 |
| 2 | hrcak.srce.hr<br>İnternet Kaynağı | %1 |
| 3 | link.springer.com<br>İnternet Kaynağı | %1 |
| 4 | www.frontiersin.org<br>İnternet Kaynağı | %1 |
| 5 | www.tandfonline.com<br>İnternet Kaynağı | %1 |
| 6 | www.ejmanager.com<br>İnternet Kaynağı | <%1 |
| 7 | www.pncongress2021.org<br>İnternet Kaynağı | <%1 |
| 8 | Erin T. Mathis, Jay Hawkins, Dominique Charlot-Swilley, Travis Spencer et al. "Mindfulness Intervention with African-American Caregivers at a Head Start Program: An Acceptability and Feasibility Study", Mindfulness, 2024<br>Yayın | <%1 |
| 9 | 9lib.net<br>İnternet Kaynağı | <%1 |
| 10 | www.mdpi.com<br>İnternet Kaynağı | <%1 |

| | | |
|---|---|---|
| 11 | Gülhan Küçük Öztürk, Nuray Şimşek. "The Effect of Motivational Interview-Based Counseling in Individuals With Amputation", Rehabilitation Nursing, 2023<br>Yayın | <%1 |
| 12 | Personnel Review, Volume 38, Issue 3 (2009-04-19)<br>Yayın | <%1 |
| 13 | www.essaysauce.com<br>İnternet Kaynağı | <%1 |
| 14 | jag.journalagent.com<br>İnternet Kaynağı | <%1 |
| 15 | integrityresjournals.org<br>İnternet Kaynağı | <%1 |
| 16 | www.kunsthochzwei.com<br>İnternet Kaynağı | <%1 |
| 17 | iiari.org<br>İnternet Kaynağı | <%1 |
| 18 | Bediye Oztas, Meltem Ugurlu, Gonul Kurt. "Fear of cancer recurrence and coping attitudes of breast cancer survivors", European Journal of Cancer Care, 2022<br>Yayın | <%1 |
| 19 | Hasibe Kadioglu, Semra Karaca, Nurhan Eren, Seher Yurt. "Development and validation of the Mental Health Promotion Scale", Perspectives in Psychiatric Care, 2021<br>Yayın | <%1 |
| 20 | ir.library.oregonstate.edu<br>İnternet Kaynağı | <%1 |
| 21 | www.magonlinelibrary.com<br>İnternet Kaynağı | <%1 |

| | | | |
|---|---|---|---|
| 22 | commons.erau.edu<br>İnternet Kaynağı | <%1 |
| 23 | www.ijfs.ir<br>İnternet Kaynağı | <%1 |
| 24 | Amir Vahedian-Azimi, Andrew C Miller, Mohammadreza Hajiesmaieli, Mari Kangasniemi et al. "Cardiac rehabilitation using the Family-Centered Empowerment Model versus home-based cardiac rehabilitation in patients with myocardial infarction: a randomised controlled trial", Open Heart, 2016<br>Yayın | <%1 |
| 25 | Metin Başoğlu, Solvig Ekblad, Sofie Bäärnhielm, Maria Livanou. "Cognitive-behavioral treatment of tortured asylum seekers: a case study", Journal of Anxiety Disorders, 2004<br>Yayın | <%1 |
| 26 | hdl.handle.net<br>İnternet Kaynağı | <%1 |
| 27 | journals.uran.ua<br>İnternet Kaynağı | <%1 |
| 28 | mail.mjltm.org<br>İnternet Kaynağı | <%1 |
| 29 | rc.library.uta.edu<br>İnternet Kaynağı | <%1 |
| 30 | tel.archives-ouvertes.fr<br>İnternet Kaynağı | <%1 |
| 31 | Piao Xue, Syed Mohamad Syed Abdullah. "A Scoping Review on Mindfulness-Based Art Therapy for University Students' Psychological Well-Being", The Arts in Psychotherapy, 2025 | <%1 |

Yayın

| | | |
|---|---|---|
| 32 | Tabea Kloos, Anna C. Elßner, Pia Berlin, Winfried Rief, Jenny Riecke. "Mobile-Based Mindfulness Intervention as Secondary Prevention of Chronic Pain: A Randomized Controlled Trial", Mindfulness, 2025<br>Yayın | <%1 |
| 33 | Tuck, Molly. "Family Matters: Family-Focused Practice in Adult Mental Health Services", The University of Manchester (United Kingdom), 2024<br>Yayın | <%1 |
| 34 | Yasemin Eskigülek, Sultan Kav. "Effect of logotherapy counseling program on chronic sorrow, dignity, and meaning in life of palliative care patients: a randomized controlled trial", Supportive Care in Cancer, 2024<br>Yayın | <%1 |
| 35 | akjournals.com<br>İnternet Kaynağı | <%1 |
| 36 | article.sapub.org<br>İnternet Kaynağı | <%1 |
| 37 | van Doorn-van Atten, Marije N.. "Nutritional Telemonitoring for Community-Dwelling Older Adults: Feasibility and Effectiveness.", Wageningen University and Research, 2021<br>Yayın | <%1 |
| 38 | Ilgın Gökler-Danışman, Merve Yalçınay-İnan, İbrahim Yiğit. "Experience of grief by patients with cancer in relation to perceptions of illness: The mediating roles of identity centrality, stigma-induced discrimination, and | <%1 |

hopefulness", Journal of Psychosocial Oncology, 2017
Yayın

| | | |
|---|---|---|
| 39 | "Psychotherapy for Cancer Patients", Springer Science and Business Media LLC, 2025<br>Yayın | <%1 |
| 40 | Guan, Ng Chong. "Management of Psychological Distress in Cancer Patients", University of Malaya (Malaysia), 2023<br>Yayın | <%1 |
| 41 | Umut Türk, Makbule Çiğdem Aydemir, Erguvan Tuğba Özel-Kizil, Sinem Civriz Bozdağ. "A Comparative Study of Anticipatory Grief in Caregivers of Patients With Alzheimer's Disease and Hematological Malignancy", Journal of the Academy of Consultation-Liaison Psychiatry, 2025<br>Yayın | <%1 |
| 42 | Volkan Demir, Selin Eryürek, Esra Savaş. "Migreni Olan Hastalarda Bilinçli Farkındalık Temelli Sanat Terapisinin Psikolojik Belirtiler ve Mutluluk Düzeylerine Etkisi: Bir Pilot Çalışma", Current Psychology, 2024<br>Yayın | <%1 |

| | | | |
|---|---|---|---|
| Alıntıları çıkart | üzerinde | Eşleşmeleri çıkar | < 5 words |
| Bibliyografyayı Çıkart | üzerinde | | |
