# Supplementary material for: Evaluating the psychosocial benefits of mindfulness-based art therapy for breast cancer patients: a preliminary study
Source: BMC Psychol. 2026 Aug 4;14:1568. doi: 10.1186/s40359-026-05154-5 (PMC13721674; doi:10.1186/s40359-026-05154-5)
Supplement: Supplementary file 2 [file 40359_2026_5154_MOESM1_ESM.docx]

Dear Editor,

We would like to thank you for the opportunity to revise our manuscript entitled “Evaluating the Psychosocial Benefits of Mindfulness-Based Art Therapy for Breast Cancer Patients: A Preliminary Study.”

In accordance with the reviewers’ and editor’s comments, we have carefully revised the manuscript. Specifically:

- **Results and conclusions:** We ensured that all results are accurately reported, toned down any potentially overstated interpretations, and fully expanded the limitations to reflect methodological and conceptual constraints.
- **STROBE guidelines:** We adhered to the STROBE statement and are submitting the completed STROBE checklist as a separate file.
- **List of abbreviations:** We included a dedicated “List of Abbreviations” section in the manuscript.
- **Proofreading:** We thoroughly proofread the entire text to correct language and grammatical errors, ensuring clarity and fluency.
- **Formatting:** The revised manuscript now contains numbered pages and continuous line numbers, and all changes are highlighted in yellow for transparency.
- **Point-by-point response:** We provide a detailed point-by-point response to each reviewer’s and editor’s comments, indicating the corresponding page and line numbers where revisions have been made.

We hope these revisions meet the journal’s requirements and expectations. We sincerely appreciate your guidance and the reviewers’ valuable feedback, which significantly improved the quality and clarity of our manuscript.

Thank you very much for considering our revised submission.

Sincerely,
Volkan Demir, PhD

on behalf of all authors
