# Supplementary material for: Evaluating the psychosocial benefits of mindfulness-based art therapy for breast cancer patients: a preliminary study
Source: BMC Psychol. 2026 Aug 4;14:1568. doi: 10.1186/s40359-026-05154-5 (PMC13721674; doi:10.1186/s40359-026-05154-5)
Supplement: Supplementary file 3 [file 40359_2026_5154_MOESM2_ESM.docx]

# STROBE Checklist with Explanations

| **STROBE Item** | **Addressed (with Explanation)** |
| --- | --- |
| 1. Title and abstract | Yes – The study design is clearly indicated in the title and abstract |
| 2. Background/rationale | Yes – The scientific background and rationale are explained in the introduction |
| 3. Objectives | Yes – The study objectives and prespecified hypotheses are presented in the introduction |
| 4. Study design | Yes – Key elements of the study design are presented at the beginning of the Methods section |
| 5. Setting | Yes – The institution, setting, and relevant dates are described in the Methods section |
| 6. Participants | Yes – Eligibility criteria, sources, and selection methods are described in the Participants section |
| 7. Variables | Yes – All variables (outcomes, exposures, predictors, confounders) are defined in the Methods section |
| 8. Data sources/measurement | Yes – Data sources and assessment methods for each variable are described in the Data Collection Tools section |
| 9. Bias | Yes – Potential sources of bias are discussed in the Limitations section |
| 10. Study size | Yes: This is a preliminary study, and the sample size (14 in the experimental group and 15 in the control group) was determined based on the pilot nature of the research and the number of patients available. A formal power analysis was not conducted; instead, the findings are presented as preliminary data. |
| 11. Quantitative variables | Yes – Handling of quantitative variables is described in the Statistical Analysis section |
| 12. Statistical methods | Yes – All statistical methods, including those used to control for confounding, are detailed |
| 13. Participants (Results) | Yes – Numbers of participants at each stage of the study are reported in the Results section |
| 14. Descriptive data | Yes – Participant characteristics (demographic, clinical, social) are presented in the Results section |
| 15. Outcome data | Yes – Outcome data are reported in tables and narrative form |
| 16. Main results | Yes – Main results are reported with statistical estimates and confidence intervals |
| 17. Other analyses | Yes – Additional/subgroup analyses are presented in the Results section |
| 18. Key results | Yes – Key results are summarized with reference to the study objectives in the Discussion |
| 19. Limitations | Yes – Limitations are discussed in detail in the Discussion section |
| 20. Interpretation | Yes – Results are cautiously interpreted in light of objectives, limitations, and related studies |
| 21. Generalisability | Yes – The generalisability (external validity) of the findings is discussed in the Discussion section |
| 22. Funding | Yes: No funding was received for this study. The research was conducted using the authors’ own resources. |
