# Supplementary material for: Evaluating the psychosocial benefits of mindfulness-based art therapy for breast cancer patients: a preliminary study
Source: BMC Psychol. 2026 Aug 4;14:1568. doi: 10.1186/s40359-026-05154-5 (PMC13721674; doi:10.1186/s40359-026-05154-5)
Supplement: Supplementary file 4 [file 40359_2026_5154_MOESM4_ESM.docx]

Dear Editor,

Thank you for the opportunity to revise our manuscript entitled “Evaluating the Psychosocial Benefits of Mindfulness-Based Art Therapy for Breast Cancer Patients: A Preliminary Study.”

We appreciate the reviewer’s constructive comment, which helped improve the clarity of the manuscript. In response to the reviewer’s suggestion, Tables 4 and 5 have been removed to avoid redundancy. The findings are now presented in a single table (formerly Table 6, now Table 4). The manuscript has been revised accordingly.

We hope that the revised version of the manuscript meets the expectations of the journal. Thank you for your time and consideration.

Sincerely,

Dr. Volkan Demir
